# The relationship between psychopathic traits and executive functioning among incarcerated men

**DOI:** 10.3389/fpsyt.2024.1524033

**Published:** 2025-01-17

**Authors:** Aleija L. Rodriguez, Corey H. Allen, J. Michael Maurer, Bethany G. Edwards, Nathaniel E. Anderson, Carla L. Harenski, Michael R. Koenigs, Kent A. Kiehl

**Affiliations:** ^1^ The Mind Research Network, Albuquerque, NM, United States; ^2^ Department of Psychiatry, University of Wisconsin-Madison, Madison, WI, United States; ^3^ Department of Psychology, University of New Mexico, Albuquerque, NM, United States

**Keywords:** psychopathy, executive function, affective deficits, inhibition deficits, antisocial traits, incarcerated sample

## Abstract

Individuals with high levels of psychopathic traits are often characterized by behaviors suggesting attenuated executive functioning (EF); however, the literature examining these two constructs have provided varied results. The current study sought to clarify the relationship between EF and psychopathic traits in a large sample of incarcerated men (*n* = 811). We utilized the Hare Psychopathy Checklist - Revised (PCL-R) and the Delis-Kaplan Executive Function System (D-KEFS) to measure psychopathic traits and EFs, respectively. D-KEFS subtests included Verbal Letter Fluency, Tower Test, Color-Word Interference Test (CWIT), and Proverbs. Regression results showed that PCL-R Factor 1 scores (measuring interpersonal and affective traits) were positively associated with verbal fluency, verbal abstraction, and verbal inhibition ability. In addition, PCL-R Facet 4 scores (measuring antisocial traits) were negatively associated with performance on inhibitory EF tasks. Our findings help further clarify the relationships between specific psychopathic traits and forms of EF, and provide potential avenues for specialized treatment or intervention approaches targeting specific psychopathic traits.

## 1 Introduction

Psychopathy is a construct characterized by callous, manipulative, and impulsive behavior (1–3). While less than 1% of the general population is estimated to meet criteria for psychopathy, the base rate increases to 15 – 25% in incarcerated settings (2). Furthermore, individuals meeting such criteria on the Psychopathy Checklist - Revised (i.e., a score of 30 or above; PCL-R) are characterized by substantially higher rates of both general and violent recidivism compared to individuals scoring lower on the PCL-R (4–6). Thus, greater understanding of individuals with elevated psychopathic traits can help potentially reduce the significant social and economic burden associated with psychopathy by providing avenues for specialized treatments.

Certain behaviors associated with psychopathy, such as impulsivity and poor behavioral controls, suggest deficits in executive function (EF), an association that has received considerable attention in the literature, albeit often with small sample sizes of incarcerated individuals (7). EF is traditionally categorized as a unified set of cognitive skills that serve diverse purposes including planning and multi-tasking (8). Though there is much debate about the full scope of EF (9), there is general agreement regarding three core components of the construct: shifting between mental tasks, updating based on new information incorporated into working memory, and inhibition of dominant or prepotent responses in favor of others (8, 9). Used successfully, EF skills can facilitate well-adjusted behavioral outcomes. One multifaceted example includes inhibiting disadvantageous behaviors based on factors including constantly-changing task requirements, re-assessment of risk, and integration of new information. In contrast, executive dysfunction has been associated with negative consequences. For example, executive dysfunction can impact daily activities that rely heavily on abilities to inhibit undesirable responses (e.g., regulating emotions to prevent an angry outburst), synchronously maintaining multiple sets of information (e.g., managing several engagements simultaneously), or updating approaches based on new information (e.g., re-organizing plans based on unexpected changes) (9). Executive dysfunction may therefore contribute to antisocial outcomes (10, 11), especially among individuals with elevated psychopathic traits.

Research that has utilized the PCL-R to examine the relationship between EF abilities and psychopathy has been equivocal. Very early conceptualizations of psychopathy suggested that the construct may be associated with improved cognitive ability compared to non-psychopaths (1). However, more recent research has suggested a negative association between EF, operationalized through performance on tests measuring planning ability and rule learning, and PCL-R total scores (12). Other literature, including a recent meta-analysis, has observed a small, but significant, negative association between psychopathic traits and inhibitory ability (13). However, other studies have not observed a significant association between psychopathic traits and EF (14–16). These and other results have cast doubt on the idea of universal EF deficits or impairments associated with psychopathy (17, 18). However, this ambiguity may be due to the fact that these previously-published studies have less frequently focused on the association between *specific* psychopathic traits (i.e., factor and facet scores) and EF, instead relying primarily on PCL-R total scores in analyses performed.

Early work with the PCL-R revealed a replicable two-factor structure (19, 20). PCL-R Factor 1 items assess interpersonal/affective psychopathic traits (e.g., glibness, callousness), and PCL-R Factor 2 items assess lifestyle/antisocial traits (e.g., irresponsibility, criminal behavior). Subsequent modeling suggested a four-facet model of the PCL-R (3, 21), separating Factor 1 into interpersonal (e.g., manipulation of others, pathological lying; Facet 1) and affective (e.g., callousness, shallow affect; Facet 2) psychopathic traits, and Factor 2 into lifestyle/behavioral (e.g., sensation seeking, impulsivity; Facet 3) and antisocial/developmental (e.g., criminal versatility, early behavior problems; Facet 4) psychopathic traits.

A number of studies have examined the relationship between PCL-R factor/facet scores and EF. In some such studies, negative associations have been observed between EF and both lifestyle and antisocial psychopathic traits (i.e., PCL-R Factor 2; 17, 22), whereas other literature has suggested that interpersonal and affective psychopathic traits (i.e. PCL-R Factor 1), particularly PCL-R Facet 2 scores measuring affective psychopathic traits, are associated with increased selective attention for task-relevant stimuli (23). Furthermore, PCL-R Factor 1 scores have been previously associated with elevated EF ability (24). While there is some literature examining psychopathic traits and EF using the PCL-R, previously-published studies have often operationalized EF using single-test (e.g., trail making task, go/nogo task) or composite operationalizations of EF (7). Therefore, a more comprehensive examination regarding the association between multiple EF domains and psychopathic traits is warranted.

The subtests included in the Delis-Kaplan Executive Function System (D-KEFS; 25) allows for the examination of several specific EFs including verbal fluency, cognitive flexibility, inhibition, cognitive set maintenance, and simultaneous processing of stimuli within the same assessment. The D-KEFS has also previously demonstrated utility in measuring EFs in incarcerated samples (26, 27). To date, only one prior study has examined the relationship between psychopathic traits and EF (operationalized using the D-KEFS) within a sample of men incarcerated in the United States (22). The authors operationally defined EF by utilizing factor analysis to derive a composite EF score from primary and secondary measures across several D-KEFS subtests, collapsing across numerous EF domains including verbal inhibition, rule learning, and cognitive flexibility (22). This study observed that this composite EF measure was negatively associated with PCL-R Factor 2 and Facet 4 scores. However, this broader operationalization of EF does not allow for more in-depth examinations regarding the association between specific EFs and psychopathic traits (22).

Given limitations in the literature regarding single-test EF operationalizations, our study aims to expand upon existing research by examining the relationship between D-KEFS subtests and PCL-R factor/facet scores in a large sample of incarcerated men. This will allow for a more nuanced understanding regarding the association between specific psychopathic traits and individual EFs. We first hypothesized that D-KEFS subtests measuring verbal EF abilities (i.e., Verbal Letter Fluency, Color-Word Interference Test [CWIT] Inhibition & Inhibition/Switching, Proverbs) would be positively associated with interpersonal/affective psychopathic traits (i.e, PCL-R Factor 1), particularly interpersonal traits (i.e., PCL-R Facet 1). This is because traits included within Facet 1 of the PCL-R, including glibness (propensity for fluid but shallow speech), superficial charm, manipulation, and pathological lying may require elevated ability for simultaneous processing, switching between tasks, or inhibiting prepotent verbal responses, domains included within the above-mentioned D-KEFS subtests. This hypothesis is bolstered by previous theorizing in the literature on such an association (24), as well as literature indicating an elevated ability to ignore interfering stimuli, maintain attentional control on goal-oriented stimuli, and switch between goal-oriented stimuli among those scoring high on psychopathy, particularly interpersonal/affective psychopathic traits (7, 28, 29). Additionally, we hypothesized that performance on EF tasks that primarily reward inhibition ability (i.e., Inhibition, Inhibition/Switching, Tower Test) would be negatively associated with lifestyle and antisocial psychopathic traits (i.e., PCL-R Facet 3 and Facet 4 scores), as such traits, including proneness to boredom and impulsivity, may reflect issues with properly inhibiting prepotent responses (7).

## 2 Method

### 2.1 Participants

Participants were recruited from adult medium- and maximum-security correctional facilities located in New Mexico and a Midwestern state, and a secure inpatient treatment facility located in a Midwestern state. Individuals were excluded if they scored below 65 on a measure of IQ (Wechsler Adult Intelligence Scale (WAIS-III; (30)), had a sub-5^th^ grade reading level (31), or met criteria for a psychotic spectrum disorder according to the Structured Clinical Interview for DSM Disorders (32, 33). The final sample consisted of 811 incarcerated adult men ranging from 19 to 65 years of age (M = 35.25, SD = 9.23) collected between 2010 and 2022. Based on racial classifications established by the National Institutes of Health, 64.9% of the sample self-identified as White, 23.3% as Black/African American, 4.9% as American Indian/Alaskan Native, 0.6% as Asian, and 6.3% as Multi-racial/Other. Regarding ethnicity, 22.1% identified as Hispanic or Latino, 76.4% as Not Hispanic or Latino, and 1.5% chose not to self-disclose their ethnicity. Participants recruited outside of New Mexico provided written informed consent according to the procedures set forth by the University of Wisconsin–Madison Human Subjects Institutional Review Board. Participants recruited in New Mexico provided written informed consent in protocols approved by the Ethical and Independent Review (E&I) Services for the Mind Research Network (a 501c3 nonprofit research institute), or by the University of New Mexico Human Research Review Committee for those consented prior to 2015.

### 2.2 Assessments and measures

#### 2.2.1 Psychopathic traits

Psychopathic traits were assessed via the PCL-R (2) using a semi-structured interview and a review of institutional records. Based on information gathered from the interview and the institutional file review, the 20 items of the PCL-R were rated zero, one, or two, reflecting the degree to which a trait was not at all present (i.e., zero), moderately present (i.e., one), or significantly present (i.e., two). PCL-R total scores can potentially range from zero to 40, and the mean PCL-R total score in the current sample was 22.3 (SD = 7.1, range: 3.2 – 38, α = 0.81) (see **Table 1** for full sample descriptive statistics). Our research group has historically completed independent double-ratings for approximately 10% of PCL-R interviews, resulting in excellent rater agreement (ICC = 0.96, *p* <.001; (34)).

**Table 1:** Sample descriptive statistics.

Variable	N	Mean	Std. Dev.	Min	Max
Age	811	35.3	9.2	19	65
PCL-R Total	811	22.3	7.1	3.2	38
PCL-R Factor 1	811	7.6	3.9	0	16
PCL-R Factor 2	811	12.4	4.0	0	20
PCL-R Facet 1	811	2.7	2.2	0	8
PCL-R Facet 2	811	4.9	2.3	0	8
PCL-R Facet 3	811	6.1	2.3	0	10
PCL-R Facet 4	811	6.4	2.6	0	10
D-KEFS Verbal Letter Fluency	811	9.1	3.2	1	18
D-KEFS Inhibition	811	9.8	3.0	1	16
D-KEFS Inhibition Switching	811	8.9	3.1	1	16
D-KEFS Tower Test	811	10.1	2.4	2	19
D-KEFS Proverbs	811	9.5	3.0	1	14

#### 2.2.2 Executive functions

EFs were assessed via the D-KEFS, which was developed using a large, representative sample that was stratified across several domains, such as education, race, ethnicity, and age (25). The D-KEFS battery comprises nine independent measures, which address a spectrum of EFs (25). Four of the nine tests from the D-KEFS were selected for this study to be consistent with previous literature on this topic (22): Verbal Letter Fluency, CWIT, Tower Test, and Proverbs. Several age-normed scaled scores were generated across all tests used. The operationalization of scores for each subtest assessed is as follows: Number of correct words provided across three trials with different target letters, with the amount of total correct responses across all three trials summed together to return a scaled score used in analyses (maximum scaled score = 19; Verbal Letter Fluency). Time to completion for two separate trials (Inhibition & Inhibition/Switching), with the time in seconds for each trial returning a scaled score used in analyses (maximum scaled scores = 19; CWIT). Sum of nine “item achievement scores” (maximum sum = 30; derived from number of moves to complete each item, correct item construction, and whether the item was built within the item-specific time limit), with this sum being the “total achievement score”, with a corresponding scaled score used in analyses (maximum scaled score = 19; Tower Test). Sum of eight “item achievement scores” (maximum sum = 32; maximum score of four for each item), which were derived from scores on accuracy (zero, one, or two) and abstraction ability (zero or two) on each item, with this sum being the “total achievement score”, with a corresponding scaled score used in analyses (maximum scaled score = 19; Proverbs; note that item responses with a zero for accuracy automatically received an “item achievement score” of zero as well, as per scoring instructions). See **Supplementary Table S1** for all correlations between PCL-R measures and D-KEFS subtests.

#### 2.2.3 Intelligence (FSIQ)

In a subset of participants included in the present sample (*n* = 642), full-scale IQ (FSIQ) was estimated using the WAIS-III (30), using the Vocabulary and Matrix Reasoning subtests (35). For the Vocabulary subtest, definitional accuracy is rated for each word (zero, one, or two), and the number of points is summed to create an age-corrected standard score. For the Matrix Reasoning subtest, the total number of correct responses is summed to create an age-corrected standard score. These standard scores are summed, and the corresponding FSIQ estimate is determined; the mean FSIQ score in the current sample was 98.9 (SD = 13.4, range: 66 – 137). See **Supplementary Table S1** for all correlations between PCL- measures and IQ.

### 2.3 Statistical analyses

For our primary hypothesis tests, multiple regression analyses were conducted using R (v. 4.3.2) and RStudio (36). Specifically, we included each D-KEFS subtest as the dependent variable (i.e., Verbal Letter Fluency, Inhibition, Inhibition/Switching, Tower Test, Proverbs) across ten separate multiple regression models, with either PCL-R factor scores (i.e., both PCL-R Factors 1 and 2 [model 1]) or facet scores (i.e., PCL-R Facets 1, 2, 3, and 4 [model 2]), along with age, as the predictor variables. Significant effects were determined at a False Discovery Rate (FDR) threshold of *p* <.05 at the individual variable level, and overall model significance was determined at a threshold of *p* <.05.

## 3 Results

### 3.1 Multiple regression analyses

#### 3.1.1 D-KEFS verbal letter fluency

Multiple regression analyses were performed to assess the relationship between D-KEFS Verbal Letter Fluency scores (measuring EFs such as verbal fluency and simultaneous processing) and PCL-R Factors (model 1) and Facets (model 2) (see **Table 2**). Both Factor and Facet models were significant: *F (3*, 807) = 8.631, *p* <.001, *R²* = .031 and *F*(5, 805) = 11.530, *p* <.001, *R²* = .067, respectively. As hypothesized, PCL-R Factor 1 (β = 0.148, *p* <.001) and PCL-R Facet 1 scores (β = 0.445, *p* <.001) were significantly associated with higher D-KEFS Verbal Letter Fluency scores, while no other Factors or Facets survived for multiple comparisons.

**Table 2:** Regressions between age, PCL-R Factors & Facets, and D-KEFS Subtests.

Variable	VLF (1)	VLF (2)	VLF *p-*values	Inh (1)	Inh (2)	Inh *p*-values	I/S (1)	I/S (2)	I/S *p-*values	TT (1)	TT (2)	TT *p-*values	Prov (1)	Prov (2)	Prov *p*-values
**Age**	-0.015 (-0.012)	-0.019 (-0.012)	.235 |.110	-0.016 (-0.011)	-0.016 (-0.011)	.172 |.150	0.033 (-0.012)	0.033 (-0.012)	.004* |.005*	-0.015 (-0.009)	-0.015 (-0.009)	.118 |.104	-0.008 (-0.012)	-0.010 (-0.011)	.503 |.384
**PCL-R Factor 1**	0.148 (-0.032)	—	<.001*	0.073 (-0.030)	—	.015*	0.081 (-0.030)	—	.008*	-0.033 (-0.024)	—	.172	0.077 (-0.030)	—	.010*
**PCL-R Factor 2**	-0.022 (-0.031)	—	.489	-0.024 (-0.029)	—	.411	-0.039 (-0.030)	—	.187	-0.038 (-0.024)	—	.104	-0.063 (-0.029)	—	.030
**PCL-R Facet 1**	—	0.445 (-0.062)	<.001*	—	0.179 (-0.059)	.002*	—	0.146 (-0.060)	.015*	—	-0.009 (-0.048)	.845	—	0.256 (-0.059)	<.001*
**PCL-R Facet 2**	—	-0.133 (-0.062)	.031	—	-0.087 (-0.058)	.135	—	-0.036 (-0.060)	.547	—	-0.042 (-0.048)	.373	—	-0.135 (-0.058)	.021
**PCL-R Facet 3**	—	-0.022 (-0.060)	.709	—	0.123 (-0.057)	.030	—	0.099 (-0.058)	.089	—	-0.070 (-0.046)	.132	—	0.041 (-0.057)	.470
**PCL-R Facet 4**	—	-0.023 (-0.046)	.611	—	-0.124 (-0.044)	.004*	—	-0.134 (-0.045)	.003*	—	-0.017 (-0.036)	.626	—	-0.135 (-0.044)	.002*
**Model F-statistic**	8.631	11.530	—	2.768	4.591	—	5.079	5.049	—	2.970	1.960	—	2.856	5.412	—
**Model *p*-value**	<.001	<.001	—	.041	<.001	—	.002	<.001	—	.031	.082	—	.036	<.001	—
**Model R²**	0.031	0.067	—	0.010	0.028	—	0.019	0.030	—	0.011	0.012	—	0.011	0.033	—

Multiple regression analyses with D-KEFS Verbal Letter Fluency (VLF), Inhibition (Inh), Inhibition / Switching (I/S), Tower Test (TT), and Proverbs (Prov) entered as dependent variables, with separate models for PCL-R Factors (1) and Facets (2), with age as a predictor variable. Variable Beta values are listed, with standard error in parentheses. Based on the inclusion of age in both models 1 and 2, the respective p-values are listed alongside each other. *Denotes significance of individual variables at a threshold of FDR p <.05.

#### 3.1.2 D-KEFS inhibition

In assessing the relationship between D-KEFS Inhibition scores (measuring verbal inhibition) and PCL-R Factors (model 1) and Facets (model 2), both models were significant: *F*(3, 807) = 2.768, *p* = .041, *R²* = .010 and *F*(5, 805) = 4.591, *p* <.001, *R²* = .028, respectively (see **Table 2**). As hypothesized, PCL-R Factor 1 (β = 0.073, *p* = .015) and Facet 1 (β = 0.179, *p* = .002) were significantly associated with higher D-KEFS Inhibition scores, while PCL-R Facet 4 (β = -0.124, *p* = .004) was also significantly associated with lower D-KEFS Inhibition scores. No other Factors or Facets survived for multiple comparisons.

#### 3.1.3 D-KEFS inhibition/switching

In assessing the relationship between D-KEFS Inhibition/Switching scores (measuring aspects of inhibition and cognitive set maintenance) and PCL-R Factors (model 1) and Facets (model 2), both models were significant: *F*(3, 807) = 5.079, *p* = .002, *R²* = .019 and *F*(5, 805) = 5.049, *p* <.001, *R²* = .030, respectively (see **Table 2**). As hypothesized, PCL-R Factor 1 (β = 0.081, *p* = .008) and Facet 1 (β = 0.146, *p* = .015) were significantly associated with higher D-KEFS Inhibition/Switching scores, while PCL-R Facet 4 (β = -0.134, *p* = .003) was significantly associated with lower D-KEFS Inhibition/Switching scores. Additionally, age (β = 0.033, *p’s* = .004,.005) was significantly associated with higher D-KEFS Inhibition/Switching scores, while no other Factors or Facets survived for multiple comparisons.

#### 3.1.4 D-KEFS tower test

In assessing the relationship between the D-KEFS Tower Test scores (measuring spatial planning and inhibition of impulsive responding) and PCL-R Factors (model 1) and Facets (model 2), model 1 was significant, *F*(3, 807) = 2.970, *p* = .031, *R²* = .011 and model 2 was moderately significant, *F*(5, 805) = 1.960, *p* = .082, *R²* = .012 (see **Table 2**). No individual variables survived for multiple comparisons.

#### 3.1.5 D-KEFS proverbs

In assessing the relationship between D-KEFS Proverbs scores (measuring verbal abstraction ability) and PCL-R Factors (model 1) and Facets (model 2), both models were significant: *F*(3, 807) = 2.856, *p* = .036, *R²* = .011 and *F*(5, 805) = 5.412, *p* <.001, *R²* = .033, respectively (see **Table 2**). As hypothesized, PCL-R Factor 1 (β = 0.077, *p* = .010) and PCL-R Facet 1 (β = 0.256, *p* <.001) were significantly associated with higher D-KEFS Proverbs scores. Additionally, PCL-R Facet 4 (β = -0.135, *p* = .002) was significantly associated with lower D-KEFS Proverbs scores, while no other Factors or Facets survived for multiple comparisons.

## 4 Discussion

The aims of the present study were to examine the relationship between specific psychopathic traits, assessed via PCL-R factor and facet scores, and EF domains, measured with individual D-KEFS subtest scores. Our results indicated that higher scores on verbal tasks of EF were associated with higher PCL-R Factor 1 and Facet 1 scores, and lower scores on inhibitory tasks of EF were associated with increased PCL-R Facet 4 scores. Overall, these results support our original hypotheses and suggest unique associations between these constructs.

In support of our first hypothesis, we observed that PCL-R Factor 1 scores (i.e., interpersonal/affective psychopathic traits) and Facet 1 scores (i.e., interpersonal psychopathic traits) were associated with higher scores on D-KEFS measures assessing verbal EF ability, including the Verbal Letter Fluency subtest, the CWIT Inhibition and Inhibition/Switching subtests, and the Proverbs subtest. These subtests associated with PCL-R Factor 1 and Facet 1 scores assess EF domains including simultaneous processing (Verbal Letter Fluency), speed of processing (Verbal Letter Fluency), verbal inhibition (CWIT), and cognitive flexibility (CWIT). PCL-R Factor 1 and Facet 1 scores were also uniquely associated with increased performance on D-KEFS Proverbs, which assesses EFs such as verbal abstract thinking, semantic integration of specific word meanings, and generalization of stimuli to multiple scenarios, in our current study.

While we observed a positive association between specific EFs and interpersonal/affective psychopathic traits, previous literature has observed a negative association between these psychopathic traits and a more general operationalization of EF (22). This may be due to the fact that this prior study examined a broader conceptualization of EF, collapsing across EF domains including verbal inhibition, rule learning, and cognitive flexibility into a single composite EF score. This is contrasted with our use of cognitive measures to examine *specific* EFs obtained from the D-KEFS, and their association with psychopathic traits.

Our results examining the relationship between PCL-R factor/facet scores and specific EFs provide support for an association between interpersonal psychopathic traits (e.g., glibness, conning and manipulative behaviors) and tasks measuring verbal EF ability. As the D-KEFS Verbal Letter Fluency test assesses and rewards fluidity rather than veracity, it would be expected that individuals scoring high on PCL-R Facet 1 would perform well on this subtest. This fluid speech may, in turn, impress other individuals through sheer volume of words—an observation previously reported among individuals scoring high on psychopathic traits (37)—rather than through meaningful speech, thereby enabling the pretense of charm that is also associated with PCL-R Facet 1.

Additionally, PCL-R Facet 1 scores were associated with improved cognitive flexibility and verbal inhibition, measured via the D-KEFS CWIT subtest. By successfully processing multiple stimuli simultaneously and quickly changing behaviors based on environmental stimuli, individuals scoring high on PCL-R Facet 1 may be characterized by increased attentional control. Indeed, individuals scoring high on PCL-R Factor 1 (which subsumes PCL-R Facets 1 and 2) have been previously associated with improved goal-oriented attentional control compared to individuals scoring lower on Factor 1 (28). Furthermore, the ability to quickly alter or inhibit one’s own behaviors to obtain a desired reaction may allow for an improved ability to manipulate other individuals. Additionally, these associations may relate to previously described positive associations between PCL-R Facet 1 scores and IQ (38, 39), whereby traits such as fluid speech and elevated processing speed assist with performance on verbal IQ tasks. This explanation is bolstered by correlation analyses within our sample, which indicate that PCL-R Facet 1 is associated with elevated performance on the WAIS-III Vocabulary subtest, which measures fluidity of speech (see **Supplementary Table S1**). In addition, previous evidence has identified cognitive flexibility as a protective factor for internalizing disorders (40, 41). It is possible that elevated cognitive flexibility associated with PCL-R Facet 1 and Factor 1 may contribute to the lower rates of internalizing disorders observed in those scoring high on PCL-R Factor 1 (42, 43). Overall, the results obtained in the current study support our initial hypothesis and may help improve our understanding of how individuals scoring high on interpersonal and affective psychopathic traits are able to manipulate and con other individuals.

In support of our second hypothesis, antisocial psychopathic traits (i.e., PCL-R Facet 4) were negatively associated with inhibition-related EF tests. For example, PCL-R Facet 4 scores, measuring antisocial psychopathic traits (e.g., poor behavioral controls and early behavioral problems) were negatively associated with D-KEFS CWIT Inhibition/Switching scores. These subtests associated with PCL-R Facet 4 assess EFs such as cognitive flexibility and inhibition (CWIT). Overall, these results support our hypotheses and suggest unique associations between these constructs. These relationships may, in turn, help contextualize the etiology of certain psychopathic traits by highlighting potential cognitive mediators of these traits.

Abnormalities associated with inhibition may contribute to the erratic lifestyle and antisocial lifestyle characteristic of individuals scoring high on psychopathy. PCL-R Facet 3 is directly related to inhibitory EFs in its measurement of traits such as impulsivity and irresponsibility, which are likely to be exacerbated by difficulties in self-regulation and disinhibition observed in executive dysfunction. The PCL-R Facet 4/EF relationship, however, may be better explained through a developmental perspective. While difficulties in impulse control and increased risk-taking often typify adolescence as a result of immature neural development, these behaviors naturally decrease throughout normative adolescent development. However, these maladaptive behavioral tendencies continue to persist in those scoring high on PCL-R Factor 2. In fact, PCL-R Facet 4 measures antisocial behavior occurring throughout an individual’s lifespan, beginning during early childhood or adolescence and *continuing* into adulthood. Specific items contained within PCL-R Facet 4 are also focused on early identification of antisocial behavior, including a history of early behavioral problems and juvenile delinquency (2). Antisocial behavior occurring during youth and adolescence may relate to inhibition-related EF deficits observed in our present results. Specifically, deficits in cognitive flexibility, cognitive set maintenance, and inhibition early in life may, in part, contribute to early antisocial behavior. Indeed, previous evidence has suggested that youth with elevated PCL: YV Facet 4 scores are characterized by error-related processing deficits, which may impair their ability to learn from mistakes (44). If left unchecked during adolescence, deficits in updating behaviors based on new information (i.e., cognitive flexibility), or difficulties with continuing advantageous behaviors based on stable external stimuli (i.e., cognitive set maintenance) may then present as impulsive or irresponsible behavior, further contributing to antisocial outcomes throughout adulthood. This interpretation is bolstered by previous evidence in the literature indicating a positive relationship between cognitive inflexibility and poor response inhibition (45). Overall, these results support our second hypothesis and suggest that deficits in specific EFs may help contribute to an impulsive, irresponsible lifestyle associated with individuals scoring high on psychopathy (46).

The knowledge gained from the present analyses carries correctional and clinical significance. Examining psychopathy with a focus on its EF corollaries could allow clinicians to use a risk-need-responsivity model for addressing specific EF-related maladaptive behaviors (e.g., impulsivity or pathological lying). For example, clinicians can utilize techniques such as dialectical behavioral therapy (DBT) to address emotion dysregulation in order to curb criminogenic risk, a proposal which has been previously suggested and implemented in correctional settings (47, 48). Understanding specific EF deficits an individual has may also inform clinicians by making them aware of potential barriers to effective treatment outcomes. For example, the thinking pattern changes sought in cognitive behavioral (CBT) and DBT paradigms benefit from an ability to shift thoughts and beliefs in favor of more adaptive perspectives over more rigid ones. Thus, individuals with cognitive inflexibility may need additional time and assistance to fully realize the benefits of these therapeutic strategies. Furthermore, previous evidence has indicated both that executive dysfunction is a significant predictor of future recidivism (49, 50), and that treatment of neurocognitive deficits, including cognitive inflexibility, contributes to positive behavioral outcomes among incarcerated individuals (51). Given that psychopathy itself is also predictive of violent outcomes and recidivism (4–6), treatment of specific EFs associated with psychopathic traits may contribute to improved institutional behavior and reduced recidivism rates among this high-risk population.

### 4.1 Study limitations and future directions

Though findings from the present student provide greater insight into the relationship between EF and psychopathic traits, some limitations remain. First, given that our findings are derived from a high-risk, incarcerated sample, it is possible that these results may not be generalizable to other samples with lower (non-clinical) levels of psychopathic traits (e.g., individuals recruited from the general community). Second, this study incorporated a sample of incarcerated adult men. Thus, we did not examine any sex differences. Future studies should examine this issue given evidence in the literature of potential sex differences in EFs (52). Third, the effect sizes regarding the associations between psychopathic traits and performance on specific D-KEFS subtests were relatively small according to their R² values (see **Table 2**). However, these small effect sizes are comparable to those found in the literature (7), suggesting their relative stability. Fourth, given that we assessed psychopathic traits using the expert-rated PCL-R, our results may not generalize to alternative instruments assessing psychopathic traits, including self-report assessments measuring traits included within the Dark Triad (e.g., Narcissism, Machiavellianism) (53). Future studies could explore whether alternative measures of psychopathic traits show similar results as reported here.

### 4.2 Conclusions

Consistent with our hypotheses, higher interpersonal/affective psychopathic traits (i.e., PCL-R Factor 1 and Facet 1 scores) were associated with improved performance on verbal EF tasks. Furthermore, higher antisocial psychopathic traits (i.e., Facet 4 scores) were associated with attenuated performance on inhibition-focused EF tasks. Our results improve upon our understanding of unique neuropsychological correlates associated with psychopathy, which can inform the management and treatment of these traits by focusing on specific cognitive mediators of maladaptive behaviors. These data also contribute to the literature by providing support for a dimensional approach to psychopathy research, supporting the position that a focus on psychopathy at the factor and facet level is an appropriate and beneficial avenue for improving our understanding regarding this construct and its cognitive correlates. The relationships and interpretations provided here suggest that specific EF strengths and weaknesses may align in unique ways, contributing to various presentations of specific psychopathic traits.

## Data Availability

The datasets presented in this article are not readily available because of the potential for personal re-identification of participants in the present sensitive population (incarcerated men). Requests to access the datasets should be directed to KKIEHL@MRN.ORG.
