# Supplementary material for: The relationship between psychopathic traits and executive functioning among incarcerated men
Source: Front Psychiatry. 2025 Jan 17;15:1524033. doi: 10.3389/fpsyt.2024.1524033 (PMC11782123; doi:10.3389/fpsyt.2024.1524033)
Supplement: Supplementary file 1 [file Table1.docx]

**Table S1. *Correlations Between Age, IQ, Psychopathic Traits, and Executive Functions***

| **Variable** | **1.** | **2.** | **3.** | **4.** | **5.** | **6.** | **7.** | **8.** | **9.** | **10.** | **11.** | **12.** | **13.** | **14.** | **15.** |
| --- | --- | --- | --- | --- | --- | --- | --- | --- | --- | --- | --- | --- | --- | --- | --- |
| **1. Age** | — |  |  |  |  |  |  |  |  |  |  |  |  |  |  |
| **2. PCL-R Total** | -.12*** | — |  |  |  |  |  |  |  |  |  |  |  |  |  |
| **3. PCL-R Factor 1** | -.10** | .82*** | — |  |  |  |  |  |  |  |  |  |  |  |  |
| **4. PCL-R Factor 2** | -.16*** | .84*** | .41*** | — |  |  |  |  |  |  |  |  |  |  |  |
| **5. PCL-R Facet 1** | -.05 | .72*** | .87*** | .36*** | — |  |  |  |  |  |  |  |  |  |  |
| **6. PCL-R Facet 2** | -.12*** | .72*** | .88*** | .36*** | .54*** | — |  |  |  |  |  |  |  |  |  |
| **7. PCL-R Facet 3** | -.16*** | .77*** | .51*** | .81*** | .41*** | .48*** | — |  |  |  |  |  |  |  |  |
| **8. PCL-R Facet 4** | -.11** | .63*** | .20*** | .85*** | .20*** | .14*** | .37*** | — |  |  |  |  |  |  |  |
| **9. VLF** | -.05 | .14*** | .17*** | .05 | .24*** | .06 | .06 | .03 | — |  |  |  |  |  |  |
| **10. Inhibition** | -.05 | .06 | .09* | .01 | .11** | .04 | .08* | -.05 | .31*** | — |  |  |  |  |  |
| **11. Inhibition Switching** | .10** | .03 | .07* | -.02 | .09* | .04 | .04 | -.08* | .30*** | .52*** | — |  |  |  |  |
| **12. Towers** | -.04 | -.09** | -.07* | -.08* | -.06 | -.07* | -.09* | -.04 | .10** | .08* | .09** | — |  |  |  |
| **13. Proverbs** | -.02 | .02 | .07 | -.04 | .12*** | .00 | .02 | -.08* | .31*** | .19*** | .20*** | .12*** | — |  |  |
| **14. WAIS-III FSIQ** | -.03 | -.02 | .03 | -.06 | .10* | -.05 | .00 | -.09* | .39*** | .30*** | .24*** | .24*** | .46*** | — |  |
| **15. WAIS-III Vocab** | -.04 | -.01 | .02 | -.05 | .12** | -.08* | .02 | -.09* | .39*** | .27*** | .24*** | .16*** | .52*** | .82*** | — |
| **16. WAIS-III Matrix** | -.05 | -.01 | .01 | -.04 | .05 | -.03 | -.02 | -.04 | .26*** | .22*** | .17*** | .24*** | .27*** | .84*** | .39*** |

*Note:* **p* < .05, ***p* < .01, ****p* < .001
